# Comparative analysis of circulating dendritic cell subsets in patients with atopic diseases and sarcoidosis

**DOI:** 10.1186/1465-9921-14-29

**Published:** 2013-03-04

**Authors:** Yumeko Hayashi, Yoshiki Ishii, Mitsumi Hata-Suzuki, Ryo Arai, Kazuyuki Chibana, Akihiro Takemasa, Takeshi Fukuda

**Affiliations:** 1 Department of Pulmonary Medicine and Clinical Immunology, Dokkyo Medical University, Kitakobayashi 880, Mibu, Tochigi, 321-0293, Japan

**Keywords:** Dendritic cells, Peripheral blood, Sarcoidosis, Myeloid DC (mDC), CD1a, CD141

## Abstract

**Background:**

Dendritic cells (DCs) are professional antigen-presenting cells that play a crucial role in the initiation and modulation of immune responses. Human circulating blood DCs are divided into two major subsets: myeloid DCs (mDCs); and plasmacytoid DCs (pDCs). Furthermore, mDCs are subdivided into two subsets: Th1-promoting mDCs (mDC1s); and Th2-promoting mDCs (mDC2s). Although CD1a, CD1c, and CD141 are generally used for classifying mDC subsets, their adequacy as a specific marker remains unclear. We performed this study to compare circulating mDC, pDC, mDC1, and mDC2 subsets between Th1- and Th2-mediated diseases using CD1a and CD141, and to analyze the adequacy of CD1a and CD141 as a marker for mDC1s and mDC2s, respectively.

**Methods:**

Thirty patients with sarcoidosis, 23 patients with atopic diseases, such as atopic bronchial asthma, and 23 healthy subjects as controls were enrolled in this study. Peripheral blood DC subsets were analyzed with flow cytometry according to expressions of CD11c, CD123, CD1a, and CD141. For functional analysis, we measured interleukin (IL) 12p40 levels produced by the sorted mDC subsets.

**Results:**

The sarcoidosis group showed decreased total DC (P < 0.05) and mDC counts (P < 0.05) compared to controls. The atopy group showed decreased CD1a^+^mDC count (P < 0.05), and increased CD1a^-^mDC count (P < 0.05) compared to controls. CD141^+^mDC count in the atopy group was higher than controls (P < 0.05). Sorted CD1a^+^mDCs produced higher levels of IL-12p40 than CD1a^-^mDCs (P = 0.025) and CD141^+^mDCs (P = 0.018).

**Conclusions:**

We conclude that decreased count of CD1a^+^mDC and increased count of CD141^+^mDC may reflect the Th2-skewed immunity in atopic diseases. The results of IL-12 levels produced by the sorted mDC subsets suggested the adequacy of CD1a and CD141 as a marker for mDC1 and mDC2, respectively, *in vivo*.

## Background

Dendritic cells (DCs) are professional antigen (Ag)-presenting cells (APCs) that originate from bone marrow and play crucial roles in the initiation and modulation of appropriate immune responses by linking innate to adaptive immune response [1,2]. Immature DCs are recruited from the blood circulation to peripheral organs, where they continuously sample the environment for foreign substances. These cells are able to take up and process antigens, and immature DCs develop into matured DC during this process with the upregulated expressions of major histocompatibility complex (MHC) and costimulatory molecules in inflammatory microenvironments [3]. Subsequently, mature DCs migrate into secondary lymphoid organs and present the processed antigens to naïve T cells for the generation of effector T cells and initiation of adaptive immune responses.

Human blood DCs comprise ~1% of peripheral blood mononuclear cells and have been classically defined as Ag-presenting leukocytes that lack other markers of leukocyte lineages (CD3, 14, 16, 19, 20, and 56) and express high levels of MHC class II (HLA-DR) molecules [4]. CD11c^+^ myeloid DCs (mDCs) and CD123^+^ plasmacytoid DCs (pDCs) represent the two major DC subsets [5], with each playing distinct and complementary roles in the induction of immune responses. Acting as strong APCs, mDCs are efficient in the uptake, processing, and presentation of foreign antigens. Following Toll-like receptor stimulation, mDCs produce tumor necrosis factor α, and matured mDCs produce proinflammatory cytokines such as interleukin (IL)-12. Conversely, pDCs are less effective in these processes and mainly known for their function in antiviral immunity and rapid production of type I interferon.

DCs are also divided into functional subtypes as a Th1-promoting subtype (DC1s) and a Th2-promoting subtype (DC2s). In the past decade, mDCs and pDCs have been thought to represent DC1s and DC2s, respectively [6,7]. However, recent studies have led to a new theory that both DC1s and DC2s differentiate from mDCs, and in some reports, the terms mDC1 and mDC2 are used to describe the mDC subsets which promote Th1 response and Th2 response, respectively. In fact, mDC1s can be generated by cultivation of monocytes with granulocyte macrophage colony-stimulating factor (GM-CSF) and IL-4, whereas mDC2s can be generated by cultivation of monocytes with IL-3 and IL-4 [8,9].

Although distinct markers of DC1 and DC2 have not yet been fully established, CD1c (blood DC antigen (BDCA)-1) and CD141 (BDCA-3) are generally used as markers of mDC1 and mDC2, respectively [10-12]. In a previous study, Th1-inducible DC subtype differentiated from peripheral monocytes by cultivation with GM-CSF expressed higher levels of CD1a than CD1c [9]. Based on these data, we use CD1a as a better marker of mDC1s. In contrast, the adequacy of CD141 as a marker for mDC2 remains controversial [13]. It is known that the expression of CD141 is not specific on mDCs, but also on pDCs [14]. Detailed functional analysis of CD1a^+^mDCs and CD141^+^ mDCs has yet to be fully discussed.

We therefore conducted a study to analyze peripheral blood DC subsets, including mDC1 and mDC2, using CD11c, CD123, CD1a and CD141, in patients with sarcoidosis as a Th1-mediated disease and atopic diseases as Th2-mediated diseases compared to healthy controls. In addition, we measured and compared levels of IL-12p40 produced by sorted mDC subsets to confirm the adequacy of CD1a and CD141 as a specific marker for circulating blood mDC1 and mDC2 subsets, respectively.

## Methods

### Study subjects

Patients who were referred to our hospital for suspected sarcoidosis or allergic bronchial asthma with or without atopic dermatitis and allergic rhinitis from 2009 to 2011 were enrolled in this study. In all the patients with sarcoidosis, the diagnosis was made from a biopsy obtained either from lung or lymph nodes and showing non-caseating granulomas, and was made in accordance with Japanese diagnostic criteria [15]. Patients who received any steroid or immunosuppressive treatments were excluded. Clinical stage was classified according to Wurm’s radiological staging [16,17]. Patients in the atopy group were defined as having a history of non-treated allergic bronchial asthma and/or atopic dermatitis and/or allergic rhinitis, with positive radioallergosorbent test (RAST; ≥class 2) to house dust mite (HDM). Healthy volunteers with negative RAST to HDM (class 0) were enrolled as the control group. To exclude the influence of smoking on peripheral DC populations, we chose only the subjects who were never-smokers or ex-smokers that had quitted smoking at least one year before the enrollment. All the subjects provided informed consent prior to enrollment. The study was approved by the Institutional Ethics Committee and was conducted in accordance with the ethical principles embodied in the Declaration of Helsinki.

### Flow cytometric analysis

**Figure 1:**
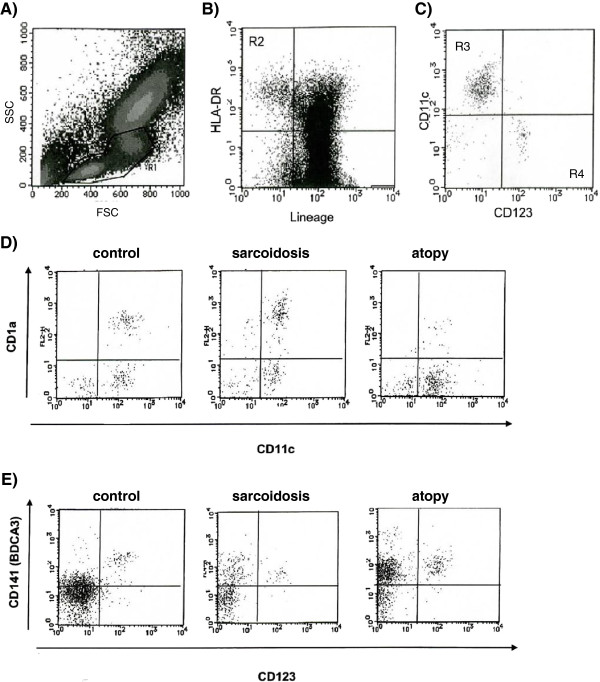
**Flow cytometric analysis of dendritic cell (DC) population from whole blood.** After gating mononucleolar cells based on side scatter (SSC) and forward scatter (FSC) **(A)**, blood DC population was identified as the lineage^-^/ HLA-DR^+^ fraction **(B)**. DCs were divided into a CD11c^+^ DC subset (mDC) and a CD123^+^ DC subset (pDC) **(C)**. mDCs (CD11c^+^DCs or CD123^-^DCs) were divided into the further subgroups based on the expression of CD1a **(D)** and CD141 **(E)**. The results presented **(D)** and **(E)** are representative of the each group.

Twenty milliliters of heparinized blood was obtained for DC isolation. Blood samples were stained with fluorescein isothiocyanate-conjugated monoclonal antibody (mAb) mixture for non-DC lineage leukocytes or lineage-negative (lin^-^) cells with specificity for CD3, CD14, CD16, CD19, CD20, and CD56, peridinin chlorophyll protein-conjugated mAbs against HLA-DR, phycoerythrin-conjugated mAbs against either the surface markers CD123 or CD1a, allophycocyanin (APC)-conjugated CD11c (BD Biosciences, San Jose, CA) or APC-conjugated CD141 (BDCA-3) (Miltenyi Biotec, Bergisch Gladbach, Germany). Following erythrocyte lysis and wash, stained leukocytes were analyzed by flow cytometry (FACS Calibur; BD Biosciences) and acquired data were analyzed using CellQuest Software (BD Biosciences). After gating mononuclear cells based on side scatter and forward scatter (Figure 1A), the blood DC population was identified as the lin^-^/ HLA-DR^+^ fraction (Figure 1B). DCs were divided into a CD11c^+^DC subset (mDCs) and a CD123^+^DC subset (pDCs) (Figure 1C). The mDC subset was further subdivided into two subsets based on the expression of CD1a (Figure 1D) and CD141 (Figure 1E). The number of total events was 200,000, and data are expressed as DC counts per 200,000 leukocytes.

### IL-12 production by sorted mDC subsets

One hundred milliliters of heparinized peripheral blood was drawn from five healthy volunteers and stained with the panel of mAbs described previously (See ‘ Flow cytometric analysis’). Peripheral blood DCs are sorted as lin^-^/HLA-DR^+^/CD11c^+^/CD1a^+^, lin^-^/HLA-DR^+^/ CD11c^+^/ CD1a^-^, lin^-^/HLA-DR^+^/CD123^-^/CD141^-^, and lin^-^/ HLA-DR^+^/CD123^-^/CD141^+^ using FACS Aria (BD Bioscience). The sorted cells were cultured in a 96-well flat-bottom tissue culture plate at 1 × 10^4^ cells/well in medium supplemented with RPMI 1640, 10% fetal bovine serum, penicillin 100 *μ*g/ mL, and lipopolysaccharide 100 ng/mL. After cultivation for 48 h, IL-12p40 levels in each supernatant were measured with purchased enzyme-linked immunosorbent assay kits (R&D Systems, Minneapolis, MN).

### Statistical analysis

Statistical analyses were performed using GraphPad Prism 5.04 (Graphpad Software, San Diego, CA). Data are summarized as mean with standard deviation (SD) or median with interquartile range (IQR) depending on distribution. Statistical differences in DC counts or ratios between groups were assessed using Kruskal-Wallis test, and subsequent post hoc analyses were performed using Dunn’s test. The paired t-test was used to compare mean values between groups. Differences were considered significant for values of P < 0.05.

## Results

### Patient characteristics

Thirty patients with sarcoidosis (13 men, 17 women; mean age, 48.3 ± 15.3 years), 23 patients with atopic diseases (16 men, 7 women; mean age 45.5 ± 14.8 years), and 23 healthy controls (10 men, 13 women; 51.7 ± 14.0 years) were enrolled in the study (Table 1).

**Table 1:** Characteristics of patients and control group

	**Sarcoidosis** **(n = 30)**	**Atopy** **(n = 23)**	**(n = 23)**
**Age: mean ± SD (range)**	**48.3 ± 15.3** **(25–72)**	**45.5 ± 14.8** **(26–70)**	**51.7 ± 14.0** **(27–67)**
**Sex** **(male/female)**	**13/ 17**	**16/ 7**	**10/ 13**
**stage I**	**8 (26.7%)**	**-**	**-**
**II**	**20 (66.7%)**		
**III**	**2 (6.7%)**		
**BA alone**	**-**	**7**	**-**
**AD alone**		**2**	
**AR alone**		**8**	
**BA + AD**		**2**	
**BA + AR**		**3**	
**AD + AR**		**1**	
**BA + AD + AR**		**0**	

BA; bronchial asthma, AD; atopic dermatitis, AR; allergic rhinitis.

### CD11c^+^DC (mDC) and CD123^+^DC (pDC) subsets

**Figure 2:**
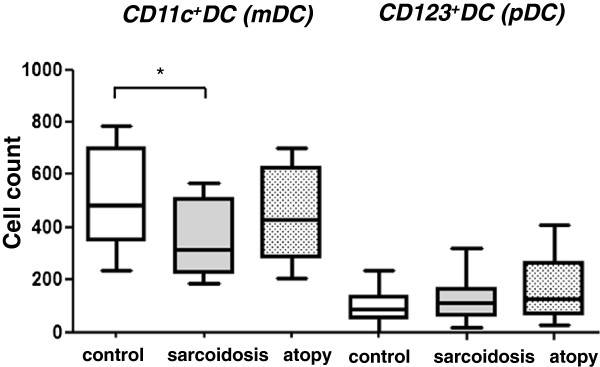
**Comparison of myeloid DC (mDC) and plasmacytoid DC (pDC) subsets.** CD11c^+^DC (mDC) subset was decreased in the sarcoidosis group (P < 0.05). CD123^+^DC (pDC) count was not different among the three groups. Data were expressed as the DC counts per 200,000 leukocytes.

All the data for the DC subset are shown in Table 2. In the sarcoidosis group, both total DC [median (IQR): 632.5 (460.0, 941.0)] and mDC [320.0 (226.0, 509.0)] counts were significantly decreased than controls [total DC: 908.0 (661.8, 1097.0) (P < 0.05), mDC: 481.0 (352.0, 700.0) (P < 0.05)] (Figure 2). pDC count was not different among the three groups. mDC count was superior to pDC count in all the three groups, and mDC/pDC-ratio did not differ among the groups (data not shown).

**Table 2:** Number of circulating DC and DC subsets in peripheral blood

	**Control**	**Sarcoidosis**	**Atopy**
**Total DC**	908.0	632.5*	1100.0
	(661.8, 1097.0)	(460.0, 941.0)	(922.6, 1279.0)
**CD11c**^**+**^**DC (mDC)**	481.0	320.0*	457.0
	(352.0, 700.0)	(226.0, 509.0)	(284.0, 628.3)
**CD123**^**+**^**DC (pDC)**	90.0	110.5	127.0
	(62.8, 143.5)	(62.5, 168.5)	(68.0, 268.0)
**CD1a**^**+**^**mDC**	351.0	238.5	215.0*
	(217.0, 462.0)	(139.8, 362.8)	(118.0, 279.0)
**CD1a**^**-**^**mDC**	92.5	79.0	205.0*
	(53.5, 144.5)	(44.5, 135.0)	(93.0, 324.5)
**CD141**^**-**^**mDC**	223.0	179.0	232.0
	(167.0, 355.5)	(126.0, 310.0)	(142.0, 299.0)
**CD141**^**+**^**mDC**	90.5	116.5	261.0*
	(52.3, 265.3)	(66.5, 166.3)	(81.5, 361.3)

Data were expressed as the DC counts per 200,000 leukocytes. Data are presented as median with interquartile range. *P < 0.05 vs control.

### CD1a^+^mDC and CD1a^-^mDC subsets

**Figure 3:**
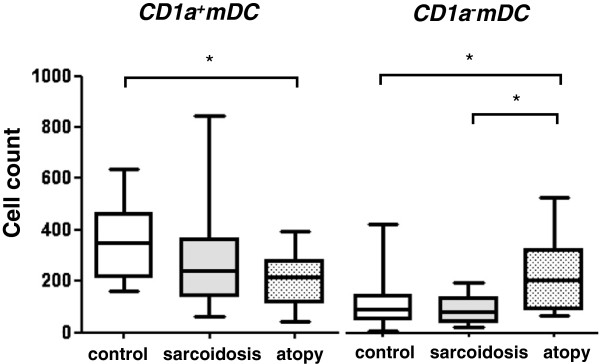
**Comparison of CD1a**^**+**^**mDC and CD1a**^**-**^**mDC subset.** CD1a^+^mDC subset was decreased in the atopy group compared to controls (P < 0.05) . CD1a^-^mDC subset was increased in the atopy group compared to both controls (P < 0.05) and the sarcoidosis group (P < 0.05). Data were expressed as the DC counts per 200,000 leukocytes.

CD1a^+^mDC count was equivalent in the sarcoidosis group: 238.5 (139.8, 362.8) and healthy controls: 351.0 (217.0, 462.0), but was significantly decreased in the atopy group: 215.0 (118.0, 279.0) (P < 0.05, Figure 3). CD1a^-^mDC count in the atopy group: 205.0 (93.0, 324.5) was greater than in both controls: 92.5 (53.5, 144.5) (P < 0.05) and the sarcoidosis group: 79.0 (44.5, 135.0) (P < 0.05).

### CD141^-^mDC and CD141^+^mDC subsets

**Figure 4:**
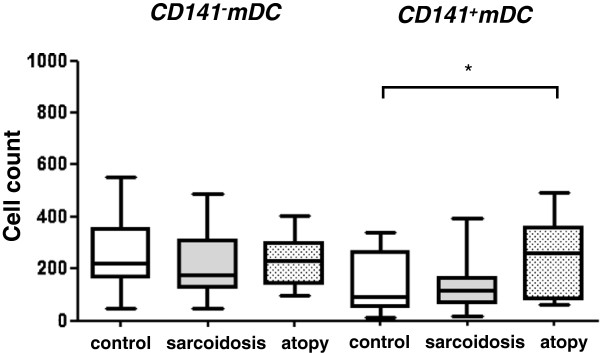
**Comparison of CD141**^**-**^**mDC and CD141**^**+**^**mDC subset.** CD141^-^mDC subset was not different among the three groups. In contrast, CD141^+^mDC in the atopy group was significantly increased than controls (P < 0.05). Data were expressed as the DC counts per 200,000 leukocytes.

No differences were seen in CD141^-^mDC count among the three groups (Figure 4). In contrast, CD141^+^mDC count was significantly increased in the atopy group compared to controls [atopy: 261.0 (81.5, 361.3), control: 90.5 (52.3, 265.3)] (P < 0.05).

### IL-12p40 levels produced by sorted mDC subsets

**Figure 5:**
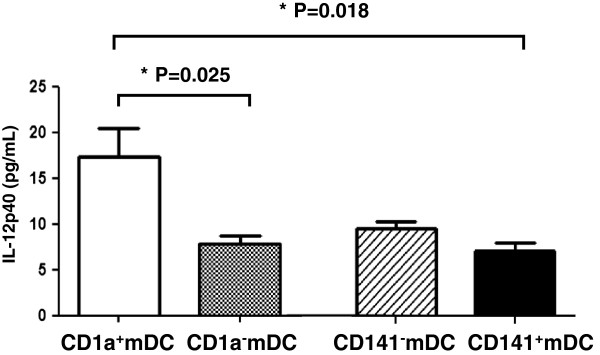
**Comparison of IL-12p40 levels produced by sorted mDC subsets.** Sorted CD1a^+^mDC produced significantly higher level of IL-12p40 compared to both CD1a^-^mDC (P = 0.025) and CD141^+^mDC (P = 0.018). There was no difference in production of IL-12p40 between CD141^-^mDC and CD141^+^mDC.

Sorted CD1a^+^mDCs produced significantly higher levels of IL-12p40 (17.3 ± 3.1 pg/mL) when compared with CD1a^-^mDCs (7.8 ± 1.9 pg/mL, P = 0.025) and CD141^+^mDCs (6.9 ± 0.8 pg/mL, P = 0.018, Figure 5). CD141^-^mDCs showed a trend toward producing higher levels of IL-12p40 (9.3 ± 0.8 pg/mL) when compared with CD141^+^mDCs (6.9 ± 0.8 pg/mL Figure 5, Table 3), but the difference was not significant (P = 0.435).

**Table 3:** Comparison of IL-12p40 levels produced by sorted mDC subsets

	**CD1a**^**+**^**mDC**	**CD1a**^**-**^**mDC**	**CD141**^**-**^**mDC**	**CD141**^**+**^**mDC**
**IL-12p40 (pg/mL)** (range)	**17.3 ± 3.1**	**7.8 ± 1.9**	**9.3 ± 0.8**	**6.9 ± 0.8**
	(10.1 – 27.2)	(5.9 – 10.8)	(7.3 – 11.8)	(4.0 – 8.5)

Data are shown as mean ± SD.

## Discussion

In this study, we have reported differences of peripheral blood DC subsets between sarcoidosis and atopic diseases using four-color flow cytometry, and have analyzed the adequacy of CD1a and CD141 as a marker for mDC1 and mDC2, respectively by the production of IL-12p40. Sarcoidosis patients showed decreased peripheral total and myeloid DC count with similar population of both CD1a^+^ mDC and CD141^+^mDC subsets compared to the control subjects. In contrast, patients with atopic diseases showed lower CD1a^+^mDC count and higher CD141^+^mDC count, which suggests the involvement of CD1a^-^mDCs and CD141^+^mDCs in Th2-polarity in atopic diseases.

In recent years, many studies have examined quantitative alternations of circulating blood DCs in patients with different pathological conditions, for example, Th1-mediated diseases such as viral infections and cancers, or Th2-mediated diseases such as bronchial asthma and atopic dermatitis [18-20]. Most investigations have focused on the proportion of mDC and pDC subsets. After a report describing two further subsets in mDCs, mDC1s as a Th1-promoting mDC subtype and mDC2s as a Th2-promoting mDC subtype [21], an increasing number of studies have been analyzing mDC1/mDC2 subsets using CD1c or CD1a, and CD141 [10,11] has been reported. CD1a and CD1c are the subgroup of CD1, which is structurally and functionally similar to MHC class I and II molecules; however, CD1 presents lipids and glycolipids rather than polypeptides on DCs. CD1 has also evolved a unique path of intracellular trafficking, processing, and loading of lipid antigens. CD141, thrombomodulin, is a glycoprotein on the surface of endothelial cells, and activates protein C as a cofactor of thrombin in the anticoagulant pathway. The immunological function of CD141 on mDCs is unknown. Relatively few studies have analyzed human peripheral blood mDC1 and mDC2 subsets. According to previous studies [10,12] performed *ex vivo*, CD1a^+^mDCs or CD1c^+^mDCs are generally accepted as representing mDC1s and CD141^+^mDCs as representing mDC2s. Hata *et al*. [9] succeeded in making mDC1s and mDC2s from freshly isolated circulating monocytes using either GM-CSF with IL-4 or IL-3 with IL-4, respectively. Furthermore, they confirmed the potential of CD1a as a marker for mDC1s through the differences in expression between mDC1 and mDC2 subsets. Based on these findings and results of previous reports [10-12], we used CD1a and CD141 in this study as a marker of mDC1s and mDC2s, respectively.

Sarcoidosis is a multisystem disorder of unknown etiology characterized by non-caseating granulomas that are composed of epithelioid cells, fibroblasts, and several immune cells such as T cells and histiocytes/macrophages. Lungs, eyes and skin are the most affected organs. In sarcoidosis, lung T cells are shown to spontaneously release high levels of IL-2 and interferon γ [22-24], and studies of T-cell clones from lung parenchyma [25] and of bronchoalveolar lavage fluid (BALF) [26-29] support the opinion of sarcoidosis as a Th1-mediated disease. Similarly, various clinical studies, genetic studies and animal models of allergic diseases support the notion that atopic diseases such as bronchial asthma, atopic dermatitis and allergic rhinitis are Th2-mediated disease.

In the present study, sarcoidosis patients showed decreased total DC and mDC counts compared to healthy controls. Previous studies have identified decreased numbers of total blood DCs and both subsets of CD11c^+^-myeloid DCs and CD11c^-^-lymphoid DCs [30]. Another report showed that sarcoidosis patients tended to show decreased numbers of mDCs [31]. Our result was similar to these previous reports. In sarcoidosis, circulating blood DCs migrate into the affected tissues, contributing to the formation of sarcoid granulomas. This migration and accumulation into local inflammatory tissues may decrease the number of DCs circulating in the blood. Ota *et al.* showed the accumulation of DCs in the lymphocyte layer of sarcoid granulomas [30].

We expected a predominance of CD1a^+^mDC count in peripheral blood in patients with sarcoidosis. Contrary to our expectation, no such differences in numbers of CD1a^+^mDCs and CD1a^-^mDCs were seen between sarcoidosis and controls. In sarcoidosis, the immunity of circulating blood does not always parallel that of locally affected organs, as seen from granuloma or BALF in lung tissue. For example, the CD4/CD8 ratio in BALF is usually high in sarcoidosis, but is not increased in peripheral blood. Given these findings, immunity of peripheral blood in patients with sarcoidosis is thought not to reflect the Th1/Th2 polarity. There are a few reports demonstrating the expression of CD1a on mDCs in the local inflammatory sites in sarcoidosis. An investigation in the BALF of inflammatory diseases showed an increase of CD1a^-^mDCs in sarcoidosis [32]. Another study of immunohistochemical investigation in muscular sarcoidosis demonstrated that CD1c^+^ mDCs scattered mainly in the lymphocyte layers of granulomas and the endomysium around the granulomas, while CD1c^+^mDCs expressed the mature DC marker CD83, but CD1a positive cells were not found by double immunostaining [33]. Since expression of surface markers on DCs is varied by the existing environment such as in organ tissue or in blood circulation, and also by the involved organs, precise evaluation is not easy. Further investigation is necessary to define the local immunity in sarcoidosis.

In the atopy group, numbers of total DC, mDC, pDC were all equivalent to controls. A previous study reported that total counts of circulating blood DCs were increased in patients with asthma [34]. Some reports have shown that allergen challenge causes a rapid decrease in the circulating mDC count [35] and an accumulation of DCs in airway epithelium [36-38] in patients with allergic asthma, while another report showed a trend toward to decreased number of circulating blood mDCs and a significant increase of pDCs in patients with atopic asthma [18]. The different disease states of the study participants may explain such contradictory results. Contrary to previous reports, which performed allergen challenge in patients with allergic asthma to invoke allergic inflammation, we examined stable atopic patients with no medication in order to exclude any influence of medications. This might have contributed to the unclear differentiation from controls. Actually, Upham *et al.* reported that decreases in circulating mDCs were most marked at 3 and 6 h post-allergen challenge, gradually returning to baseline levels [35].

In the atopy group, CD1a^+^mDC count showed a significant decrease, while CD141^+^mDC count was significantly increased compared to controls. Yerkovich *et al.* reported that after allergen challenge the constitutive expression of CD141 on mDCs was increased in atopic individuals compared to non-atopic subjects [12]. The same group of investigators has also shown that CD141^+^mDCs are associated with Th2 polarizing response, whereas CD141^-^mDCs are associated with a mixed Th1/Th2 response. On the other hands, Jongbloed *et al.* reported that CD141^+^mDCs induce superior Th1 response compared to CD1c^+^DCs [13], and the significance of CD141^+^mDCs is still in dispute. The results of the present study may support the theory that CD1^+^mDCs and CD141^+^mDCs represent mDC1s and mDC2s, respectively. The decreased mDC1s and increased mDC2s may reflect the Th2-skewed immunity in atopic disease.

When discussing the adequacy of CD1a and CD141 as a definite marker for mDC1s and mDC2s, respectively, the ability of sorted CD1a^+^mDCs and CD141^+^mDCs to induce Th1 and Th2, respectively, must be confirmed. However, few reports have examined this area, because these procedures need large amounts of peripheral blood, reducing clinical feasibility. In a previous report, monocyte-derived CD1a^+^mDCs produced high amounts of IL-12, a Th1-inducible cytokine, and induced Th1 when co-cultured with lymphocytes [10]. In our study, sorted CD1a^+^mDCs produced significantly higher levels of IL-12p40 compared to CD1a^-^mDCs. This result suggests that CD1a can offer a marker for human peripheral blood mDC1s. CD141^+^mDCs produced significantly less IL-12p40 compared to CD1a^+^mDCs but no differences in produced IL-12p40 levels were seen between CD141^+^mDCs and CD141^-^mDCs. CD141^-^mDC subset is probably composed of heterogeneous cell populations, and they are not always represent mDC2 subset. So, our results imply the possibility of CD141 as a marker for mDC2, however, further investigation is necessary to clarify the meaning of the expression of CD141 on mDCs as mDC2.

In the present study, we were unable to analyze CD1a/CD141 double positive or double negative mDCs due to technical limitations. There is almost no data about them in human peripheral blood, however, Bratke et al. showed that 45% of mDCs were CD1a positive and 78% of mDCs were CD141 positive in the analysis of BALF of never smokers [39]. It may be difficult to completely identify mDC1 and mDC2 only using CD1a and CD141. In future research, we feel it is necessary to find out the number and function of CD1a/CD141 double positive and double negative mDCs in human peripheral blood.

## Conclusions

In conclusion, the present study shows, for the first time, human circulating DC subsets including mDC1 and mDC2 comparing Th1-related to Th2-related disease. These data suggests the involvements of mDC1s and mDC2s in the Th1/Th2-polarity, and the adequacy of CD1a as a marker for mDC1 and the possibility of CD141 as a marker for mDC2.

## Abbreviations

DC: Dendritic cell; MHC: Major histocompatibility complex; GM-CSF: Granulocyte macrophage colony-stimulating factor; IL: Interleukin; BDCA: Blood dendritic cell antigen; RAST: Radioallergosorbent test; HDM: House dust mite; HLA: Human leukocyte antigen; APC: Allophycocyanin; SSC: Side scatter; FSC: Forward scatter

## Competing interests

The authors declare that they have no competing interests.

## Authors’ contributions

Conception and design: YH, YI; acquisition of data: YH, MS, RA, KC; analysis and interpretation of data: YH, MS, YI; drafting of the manuscript: YH, YI; critical revision of manuscript: YI, TF. All authors have read and approved the final manuscript.

## References

[B1] Steinman RM The dendritic cell system and its role in immunogenicity Annu Rev Immunol 1991 9 271 296 10.1146/annurev.iy.09.040191.001415 1910679

[B2] Banchereau J Steinman RM Dendritic cells and the control of immunity Nature 1998 392 245 252 10.1038/32588 9521319

[B3] Sato K Fujita S Dendritic cells-nature and classification Allergol Int 2007 56 183 191 10.2332/allergolint.R-06-139 17646733

[B4] Hart DN Dendritic cells: unique leukocyte population, which control the primary immune response Blood 1997 90 3254 3287 9345009

[B5] Ito T Liu YJ Kadowaki N Functional diversity and plasticity of human dendritic cell subsets Int J Hematol 2005 81 188 196 10.1532/IJH97.05012 15814329

[B6] Rissoan MC Soumelis V Kadowaki N Grouard G Briere F de Waal MR Liu YJ Reciprocal control of T helper cell and dendritic cell differentiation Science 1999 283 1183 1186 10.1126/science.283.5405.1183 10024247

[B7] Moser M Murphy KM Dendritic cell regulation of T_H_1-T_H_2 development Nature Immunol 2000 3 199 205 10973276 10.1038/79734

[B8] Ebner S Hofer S Nguyen VA Fürhapter C Herold M Fritsch P Heufler C Romani N A novel role for IL-3: human monocytes cultured in the presence of IL-3 and IL-4 differentiate into dendritic cells that produce less IL-12 and shift Th cell responses toward a Th2 cytokine pattern J Immunol 2002 168 6199 6207 12055233 10.4049/jimmunol.168.12.6199

[B9] Hata M Takahara S Tsuzaki H Ishii Y Nakata K Akagawa KS Satoh K Expression of Th2-skewed pathology mediators in monocyte-derived type 2 of dendritic cells (DC2) Immunol Lett 2009 126 29 36 10.1016/j.imlet.2009.07.008 19643136

[B10] Chang CC Wright A Punnonen J Monocyte-derived CD1a^+^ and CD1a^-^ DC subsets differ in their cytokine production profiles, susceptibilities to transfection, and capacities to direct Th cell differentiation J Immunol 2000 165 3584 3591 11034359 10.4049/jimmunol.165.7.3584

[B11] Dzionek A Fuchs A Schmidt P Cremer S Zysk M Miltenyi S Buck DW Schmitz J BDCA-2, BDCA-3 and BDCA-4: three markers for distinct subsets of dendritic cells in human peripheral blood J Immunol 2000 165 6037 6046 11086035 10.4049/jimmunol.165.11.6037

[B12] Yerkovich ST Roponen M Smith ME McKenna K Bosco A Subrata LS Mamessier E Wikström ME Le Souef P Sly PD Holt PG Upham JW Allergen-enhanced thrombomodulin (blood dendritic cell antigen 3, CD141) expression on dendritic cells is associated TH2-skewed immune responses J Allergy Clin Immunol 2009 123 209 216 10.1016/j.jaci.2008.09.009 18947863

[B13] Jongbloed SL Kassianos AJ McDonald KJ Clark GJ Ju X Angel CE Chen CJ Dunbar PR Wadley RB Jeet V Vulink AJ Hart DN Radford KJ Human CD141^+^ (BDCA-3)^+^ dendritic cells (DCs) represent a unique myeloid DC subset that cross-presents necrotic cell antigens J Exp Med 2010 207 1247 1260 10.1084/jem.20092140 20479116 PMC2882828

[B14] Bratke K Lommatzsch M Julius P Kuepper M Kleine HD Luttmann W Christian VJ Dendritic cell subsets in human bronchoalveolar lavage fluid after segmental allergen challenge Thorax 2007 62 2 168 175 10.1136/thx.2006.067793 16928719 PMC2111237

[B15] JASOG criteria, report of research project ‘diffuse lung Diseases’ supported by ministry of public welfare 1989 160 162 in Japanese

[B16] Wurm K Reindell H Heilmyer L Der Lun fenboek in Rontgebild 1958 Stuttgart: George Thieme

[B17] ATS/WASOG Statement of sarcoidosis Sarcoid Vasc Diffuse Lung Dis 1999 16 147 173 10560120

[B18] Matsuda H Suda T Hashizume H Yokomura K Asada K Suzuki K Chida K Nakamura H Alternation of balance between myeloid dendritic cells and plasmacytoid dendritic cells in peripheral blood of patients with asthma Am J Respir Crit Care Med 2002 166 1050 1054 10.1164/rccm.2110066 12379547

[B19] Perrot I Blanchard D Freqymond N Issac S Guilbert B Pacheco Y Lebecque S Dendritic cells infiltrating human non-small cell lung cancer are blocked at immature stage J Immunol 2007 178 5 2763 2769 17312119 10.4049/jimmunol.178.5.2763

[B20] Almeida M Cordero M Almeida J Orfao A Different subsets of peripheral blood dendritic cells show distinct phenotypic and functional abnormalities in HIV-1 infection AIDS 2005 19 3 261 271 15718836

[B21] Autissier P Soulas C Burdo TH Evaluation of a 12-color flow cytometry panel to study lymphocyte, monocyte, and dendritic cell subsets in humans Cytometry A 010 77 410 419 20099249 10.1002/cyto.a.20859 PMC11742174

[B22] Pinkston P Bitterman P Crystal R Spontaneous release of interleukin-2 by lung T lymphocytes in active pulmonary sarcoidosis N Engl J Med 1983 308 793 800 10.1056/NEJM198304073081401 6601235

[B23] Robinson B Mclemore T Crystal R Gamma interferon is spontaneously released by alveolar macrophage and lung T lymphocytes in patients with pulmonary sarcoidosis J Clin Invest 1985 75 1488 1495 10.1172/JCI111852 3923038 PMC425487

[B24] Prior C Knight R Herold M Ott G Spiteri M Pulmonary sarcoidosis: patterns of cytokine release *in vitro* Eur Respir J 1996 9 47 53 10.1183/09031936.96.09010047 8834333

[B25] Bümer I Zissel G Schlaak M Müller-Quernheim J Th1/Th2 cell distribution in pulmonary sarcoidosis Am J Respir Cell Mol Biol 1997 16 171 177 9032124 10.1165/ajrcmb.16.2.9032124

[B26] Hoshino T Itoh K Gouhara R Yamada A Tanaka Y Ichikawa Y Azuma M Mochizuki M Oizumi K Spontaneous production of various cytokines except IL-4 from CD4+ T cells in the affected organs of sarcoidosis patients Clin Exp Immunol 1995 102 399 405 7586698 10.1111/j.1365-2249.1995.tb03797.x PMC1553396

[B27] Garlepp M Rose A Dench J Robinson B Clonal analysis of lung and blood T cells in patients with sarcoidosis Thorax 1994 49 577 585 10.1136/thx.49.6.577 8016795 PMC474948

[B28] Walker V Braum W Menz G Braum P Schwarz F Hansel T Villiger B Activated T cells and cytokines in bronchoalveolar lavages from patients with various lung diseases associated with eosinophilia Am J Respir Crit Care Med 1994 150 1038 1048 7921434 10.1164/ajrccm.150.4.7921434

[B29] Moller D Forman J Liu M Nobel P Greenlee B Vyas P Holden D Forrester J Lazarus A Wysocka M Trinchieri G Karp C Enhanced expression of IL-12 associated with Th1 cytokine profiles in active pulmonary sarcoidosis J Immunol 1996 156 4952 4960 8648147

[B30] Ota M Amakawa R Uehira K Ito T Yagi Y Oshiro A Date Y Oyaizu H Shigeki T Ozaki Y Yamaguchi K Umeura Y Yonezu S Fukuhara S Involvement of DCs in sarcoidosis Thorax 2004 59 408 413 10.1136/thx.2003.006049 15115868 PMC1746987

[B31] Mathew S Bauer KL Fischoeder A Bhardwaj N Oliver SJ The anergic state in sarcoidosis is associated with diminishied dendritic cell function J Immunol 2008 181 746 755 18566441 10.4049/jimmunol.181.1.746 PMC2593870

[B32] Lommatzsch M Bratke K Bier A Julius P Kuepper M Luttmann W Virchow JC Airway dendritic cell phenotypes in inflammatory diseases of the human lung Eur Respir J 2007 30 878 886 10.1183/09031936.00036307 17626112

[B33] Tateyama M Fujihara K Itoyama Y Dendritic cells in muscle lesions of sarcoidosis Hum Pathol 2011 42 340 346 10.1016/j.humpath.2010.07.011 21111453

[B34] Spears M Mcsharry C Donnelly I Jolly L Branningam M Thomson J Lafferty J Chaundhuri R Shepherd M Cameron E Thomson NC Peripheral blood dendritic cell subtypes are significantly elevated in subjects with asthma Clin Exp Allergy 2011 41 665 672 10.1111/j.1365-2222.2010.03692.x 21338429

[B35] Upham JW Denburg JA O’Byrne PM Rapid response of circulating myeloid dendritic cells to inhaled allergen in asthmatic subjects Clin Exp Allergy 2002 32 818 823 10.1046/j.1365-2222.2002.01375.x 12047425

[B36] Bellini A Vittori E Marini M Ackerman V Mattoli S Intraepithelial dendritic cells and selective activation of Th2-like lymphocytes in patients with atopic asthma Chest 1993 103 997 1005 10.1378/chest.103.4.997 8131514

[B37] Jahnsen FL Moloney ED Hogan T Upham JW Burke CM Holt PG Rapid dendritic cell recruitment to the bronchial mucosa of patients with atopic asthma in response to local allergen challenge Thorax 2001 56 823 826 10.1136/thorax.56.11.823 11641504 PMC1745967

[B38] Möller GM Overbeek SE Van Helden-Meeuwsen CG Van Haarst JM Prens EP Mulder PG Postma DS Hoogsteden HC Increased numbers of dendritic cells in the bronchial mucosa of atopic asthmatic patients: down regulation by inhaled corticosteroids Clin Exp Allergy 1996 26 517 524 10.1111/j.1365-2222.1996.tb00571.x 8735863

[B39] Bratke K Klug M Bier A Julius P Kuepper M Virchow C Lammatzsch M Function- associated surface molecules on airway dendritic cells in cigarette smokers Am J Respir Cell Mol Biol 2008 38 655 660 10.1165/rcmb.2007-0400OC 18203971

